# Mirror-backed Dark Alumina: A Nearly Perfect Absorber for Thermoelectronics and Thermophotovotaics

**DOI:** 10.1038/srep19984

**Published:** 2016-01-28

**Authors:** Mohamed Farhat, Tsung-Chieh Cheng, Khai. Q. Le, Mark Ming-Cheng Cheng, Hakan Bağcı, Pai-Yen Chen

**Affiliations:** 1 Division of Computer, Electrical, and Mathematical Sciences and Engineering, King Abdullah University of Science and Technology (KAUST) Thuwal 23955-69100, Saudi Arabia; 2 Department of Mechanical Engineering, National Kaohsiung University of Applied Science (KUAS), Kaohsiung 80778, Taiwan, Republic of China; 3 Department of Electrical Engineering, University of Minnesota, Duluth, Minnesota 55812, USA; 4 Department of Electrical and Computer Engineering, Wayne State University, Detroit, Michigan 48202, USA

## Abstract

We present here a broadband, wide-angle, and polarization-independent nearly perfect absorber consisting of mirror-backed nanoporous alumina. By electrochemically anodizing the disordered multicomponent aluminum and properly tailoring the thickness and air-filling fraction of nanoporous alumina, according to the Maxwell-Garnet mixture theory, a large-area dark alumina can be made with excellent photothermal properties and absorption larger than 93% over a wide wavelength range spanning from near-infrared to ultraviolet light, i.e. 250 nm–2500 nm. The measured absorption is orders of magnitude greater than other reported anodized porous alumina, typically semi-transparent at similar wavelengths. This simple yet effective approach, however, does not require any lithography, nano-mixture deposition, pre- and post-treatment. Here, we also envisage and theoretically investigate the practical use of proposed absorbers and/or photothermal converters in integrated thermoelectronic and/or thermophotovoltaic energy conversion devices, which make efficient use of the entire spectrum of ambient visible to near-infrared radiation.

Conversion of ambient electromagnetic radiation, such as sunlight, blackbody radiation and radio waves from electronic transmitters, into electricity can be realized using a variety of techniques, such as photovoltaics (PV)^1^,^2^,^3^, thermoelectrics (TE)^4^, thermophotovoltaics (TPV)^5^,^6^,^7^, thermionic conversion (TC) (or thermoelectronics)^8^,^9^,^10^,^11^, and rectification of electromagnetic waves^12^,^13^,^14^. Among these, TC and TPV techniques are considered as highly efficient techniques that harvest photon energy from sunlight and thermal radiation within a broad photon energy (from infrared (IR) to ultraviolet (UV) wavelengths) and transduce them into thermal energy, followed by a direct conversion process from heat to electricity. Ideally, TC and TPV solar cells can overcome fundamental challenges for conventional PV solar cells, through the efficient use of the entire solar spectrum^5^,^6^,^7^,^8^,^9^,^10^. A thermionic converter illustrated in Fig. 1(a) is based on a fairly simple vacuum microdiode, where the hot electrode (emitter) heated by focused solar irradiation or thermal radiation can thermionically emit electrons over a potential barrier to a cooler electrode (collector), thus producing the useful electric power output^8^,^9^,^10^,^11^. A TPV cell illustrated in Fig. 1(b) is operated in a somewhat more complex manner: the absorbed heat is first converted into a narrowband thermal radiation by a frequency-selective emitter, and then the re-radiated electromagnetic energy with wavelength matched to the bandgap of PV receivers is converted into electrical energy without losses from thermalization or Joule heating^5^,^6^,^7^. In general, TC and TPV solar panels require extremely large optical concentrators along with bulky mechanical trackers to provide reasonably high temperatures. The high pumping irradiance, necessary for efficient energy conversion, makes the practice of TC and TPV devices particularly challenging in terms of cost, efficiency and reliability. It is believed that significant improvements in those areas can be made by designing a nearly perfect electromagnetic energy absorber that can achieve broadband, wide-angle and polarization-independent absorption^15^,^16^,^17^,^18^,^19^,^20^,^21^,^22^,^23^,^24^,^25^,^26^,^27^, as well as excellent photothermal properties.

With the rapid advent of nanotechnology, design of highly-efficient and compact antireflection coatings or surface absorbers has become viable using nanophotonic techniques: photonic nanostructures^18^,^19^,^20^,^21^,^22^,^23^,^24^,^25^,^26^,^27^,^28^,^29^, photonic crystals^30^,^31^, and metamaterials^15^,^16^,^17^. Aiming to facilitate the use of TC and TPV energy conversion devices, here we develop a simple and cost-effective chemical route to prepare a high-performance, large-area absorber constructed using a nanoporous alumina film on top of the commercial 6061-T6 aluminum (Al) substrate. This absorber can exhibit absorption larger than 93% over a broad range of wavelengths (250 nm–2500 nm) and incident angles (0°–90°) for both transverse electric (TE) and transverse magnetic (TM) polarizations. Such performance are comparable or better than previous designs^18^,^19^,^20^,^21^,^22^,^23^,^24^,^25^,^26^,^27^,^28^. However, the proposed approach may have advantages over conventional lithographic nanostructures in terms of high throughput, low cost, large patterned areas, and ability to be integrated in thermoelectronics and thermophotovotaics systems. We should also note that this mirror-backed structure can have dual functions. Except for acting as an efficient absorber/photothermal converter that absorbs electromagnetic energy and converts it into heat by raising the substrate temperature, the back-side metal surface can be nano-engineered to realize efficient thermionic electron emitters or thermal re-radiators in TC and TPV devices.

Micro/nano-porous metal oxides, particularly anodic aluminum oxide (AAO), have a long list of applications in optical, chemical, and material sciences and engineering^32^,^33^,^34^,^35^,^36^,^37^,^38^. Typically, AAO membranes are quasi-transparent and have well-arranged periodic air pores. A self-assembled AAO porous layer has been widely used as the template for the growth of uniform, periodic, and well-aligned nanotubes and nanowires^39^ and the formation of nanoparticle arrays^40^, the basis for photonic bandgap structures^41^,^42^, and the anti-reflection coating for light trapping^43^. In these applications, the porous AAO membrane, being transparent in the IR and optical frequencies, was prepared using high-quality aluminum treated chemically by sulfuric acid adonization at low current density and moderate DC bias voltage during the electrochemical thinning. For certain applications, which require high optical absorption, lossy colloidal nanoparticles, e.g. metallic nanospheres^33^,^44^ or carbon nanotubes^45^, are typically deposited onto the AAO template for enhancing the light absorption. In this work, we propose a simple yet effective method to develop large-area *dark* AAO membranes with ultrahigh absorption in a wide wavelength range, without any post-treatment or deposition of nano-mixtures. Specifically, the commercial 6061-T6 aluminum sheet containing various alloying elements, such as magnesium, iron, and silicon impurities, was treated by the high-voltage sulfuric acid hard anodizing^46^,^47^. The 6061-T6 aluminum alloy has been extensively used in many applications, for its several advantages, including low cost, lightweight, high melting point, and acceptable strength. Since 6061-T6 aluminum has sufficiently high concentration of diffused impurities and alloying elements, its optical absorption is much greater than purified aluminum. This can be explained by the increased electron collision rate (electron-electron and electron-phonon) in solids, due to the high density of impurities and crystal defects. In addition, the high-voltage anodization, together with the disordered multicomponent crystal structure of 6061-T6 aluminum, may result in an amorphous nanoporous alumina layer on the surface of anodized aluminum. The effective refractive index of the synthesized AAO is therefore complex-valued, with a relevant imaginary part responsible for optical losses. Consequently, the proposed high-voltage sulfuric acid anodizing applied to the 6061-T6 aluminum substrate allows a simple, rapid and cost-effective fabrication of highly efficient absorbers for visible and near-infrared radiation.

## Results

### Optically Lossy Nanoporous Alumina Films

In this work, a set of optically lossy porous alumina (i.e. AAO) films on top of aluminum substrate were fabricated. A 2cm×2cm commercial 6061-T6 aluminum alloy was treated by a standard electrolytic polishing. Then, the aluminum sheet was immersed in a 0.3 M sulfuric acid solution for hard anodizing. During the hard anodizing process, the DC bias was fixed to 30 V and the medium temperature was controlled by a precise cooling system. We prepared three species under different process temperatures: 0 °C, 5 °C, and 10 °C. The hard anodizing process was performed for 1 hour, followed by the standard deionized water rinse and blow dry. Due to the high concentration of sulfuric acid and the high electrostatic biasing field in the hard anodizing process, the active surface oxidation results in rapid Joule heating and imposes morphological damages to the surface, which is quite sensitive to the process temperature. To study the effect of electrostatic biasing, we also prepared two species under higher DC bias voltage of 40 V and 50 V, at the process temperature of 10 °C. Conventional AAOs are made based on the costly high-purity (99.997%) aluminum foils^33^, which yield an optically transparent porous alumina that hardly absorbs infrared and visible light. Our approach, although based on similar electrochemical process, utilized the high-impurity alloy that is naturally lossy in infrared and visible regions, as its collision rate in the fitted Drude model is ~10 times larger than pure aluminum. The resulting alumina presents an exotic dark color, as a clear evidence of broadband absorption of visible light.

Table 1 summarizes the thickness and air-filling ratio (ratio of air in this porous medium) of synthesized dark alumina under different anodizing conditions. The morphology, air-filling fraction, and thickness of nanoporous alumina film are determined by several factors, including the electrolyte concentration, the temperature and the DC bias in the anodizing process. Figure 2(a) shows the corresponding CCD images for different AAO-covered, anodized aluminum in Table 1; the untreated aluminum is also shown here for a fair comparison. It is surprising to see that an AAO coating, i.e. nanoporous alumina, can turn the highly reflective surface of aluminum into dramatically dark, by absorbing most of the impinging visible light. We also note that greater opacity of AAO-coated aluminum is obtained by raising the process temperature, due to the increased thickness and air-filling ratio of AAO, which will be explained in the following. Figure 2(b,c) show the top-view and cross-sectional scanning electron microscope (SEM) images of sample 4 in Fig. 2(a). We found that unlike those conventional AAO membranes having crystal-like hexagonal periodicity, the synthesized AAO film exhibits irregularly distributed nanopores with inconsistent sizes. The cross-sectional SEM image also reveals that nanopores have high aspect ratio, and are misaligned and randomly orientated in the vertical direction.

### Infrared and Optical Absorption

Here we demonstrate that the concept and feasibility of broadband and wide-angle absorption enabled by the mirror-backed mesoporous metal oxide (MMO), as shown in Fig. 3(a). The surface-anodized aluminum alloys studied here, i.e. AAO plus aluminum, could be a representative structure, which, however, can be readily, prepared using the simple and cost-effective electrochemical process^48^. For an N-phase composite medium consisting of randomly distributed subwavelength inclusions, its macroscopic effective permittivity *ε*_eff_ can be analytically derived from the Maxwell-Garnett theory^49^ as:


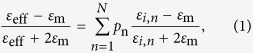


where *p*_*n*_ and *ε*_i,n_ are the volume fraction and relative permittivity of the *N*-th inclusion in this mixture, and *ε*_m_ is the relative permittivity of host matrix. Consider the two-phase mesoporous material in Fig. 2(b,c): irregularly-distributed, subwavelength nanopores filled with air (i.e. *ε*_m_ = 1) and embedded in a metal oxide host matrix of relative permittivity *ε*_MMO_, the effective relative permittivity is given by:


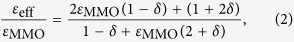


where *δ* is the volume fraction of air nanopores. Since air nanopores with average diameter less than 20 nm are deeply subwavelength, this MMO medium can be treated as a homogenous medium of relative permittivity *ε*_eff_. By properly tailoring the complex effective refractive index (or optical impedance) of metal oxide, the maximum light absorption can be obtained in certain conditions. Here we used a transmission line (TL) approach^18^,^50^ depicted in Fig. 3(b) to model the light scattering from such a mirror-backed composite medium, i.e. MMO. The TL approach is particularly suitable for studying the plane waves incident on the bulk homogeneous medium, without the excitation of higher-order diffracted Floquet modes. The free-space region and metal substrate are modeled as semi-infinite TLs and the MMO region is treated as a TL segment of length *l*. For a given angle of incidence *θ* with respect to the surface normal direction [see Fig. 3a], the effective free-space wave number is 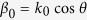, i.e., the longitudinal component of the impinging wave vector, the characteristic impedance per unit length are 
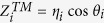
 and 
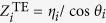
 of the *i*-th medium for TM and TE incident waves, where 
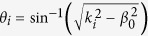
, 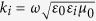, and 
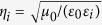
, are the propagation angle, wave vector, and intrinsic impedance of the *i*-th medium, *ω* is the radian frequency, *ε*_i_ is the relative permittivity of the *i*-th medium, and *ε*_0_ and *μ*_0_ are vacuum permittivity and permeability. According to the TL model in Fig. 3(b), the reflection coefficient at the input of MMO surface can be derived as:


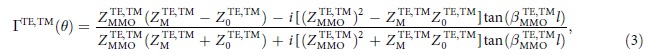


and the total absorption of this system is given by


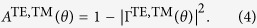


The relative permittivity of metal substrate follows a Drude-type dispersion^51^,^52^, 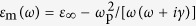, where *ω*_p_ is the plasma frequency and *γ* is the collision rate. For aluminum, parameters extracted from experimental data are 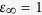, 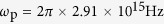, and 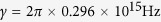. We note that the value of *γ* used here is 9.5 times larger than that of the best quality purified aluminum, owning to the existence of various residual impurities and alloying elements in 6061-T6 aluminum, which would increase the material loss that depends on the volume fraction of air and lossy alumina.

Figure 3(c,d) report the measured spectral absorption for 6061-T6 aluminum and various anodized alumina in Table 1. The UV-VIS-NIR spectrophotometer (integrating sphere detector) shown in the Fig. 3(a) was used here to rigorously characterize the light absorption of samples in a wide wavelength range of 250 nm–2500 nm. The measured average absorption by spectrophotometer over all angles of illumination is defined as:


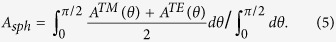


The experimental data (solid lines) was verified by the theoretical results (dots) obtained from the TL model, and excellent agreements are obtained for all cases; here realistic physical parameters in Table 1 were used in our theoretical calculations. The refractive index of the MMO layer (AAO here) is 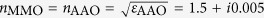
34,35, valid at wavelengths of interest. We note that the imaginary part of *n*_AAO_ is significantly larger than that of most AAO membranes and is expected to enhance the absorption of incident radiation. The TL approach is an effective tool to design, analyze and optimize the scattering and absorption of mirror-backed MMO structures. From Fig. 3(c,d), it is evident that the 6061-T6 aluminum is more lossy than the high-quality aluminum (e.g. those prepared by physical vapor deposition or sputtering^51^,^52^), with almost ten times larger collision rate in the fitted Drude model. We note that the interband transition of aluminum is observed at the wavelength *λ* = 879.4 nm^51^,^52^, which introduces extra absorption and is not considered in the Drude model. From Fig. 3(c,d), we found that hard anodizing can dramatically increase the absorption of aluminum, and the absorption increases with increasing the anodizing temperature and DC bias. The sample 4 of larger AAO thickness and higher porosity exhibits a 93.5% average absorption in the UV/visible and a 92.3% average absorption in the near-infrared region. Such high absorption can be attributed to the optical impedance matching, for which a nanoporous AAO thinfilm can have real-part effective impedance (or refractive index) close to that of free space region (background medium). Based on the mixture law in Equation (2), the effective impedance of AAO can be tailored at will by designing the volume fraction of air pores, and a close-to-zero reflection may be obtained when AAO and incident medium have the equal value of characteristic impedance. Figure 4(a,d) show the theoretical contours of absorptions for a mirror-backed porous alumina at different wavelengths: (a) 500 nm, (b) 1000 nm, (c) 1500 nm, and (d) 2000 nm, varying the porosity and thickness; all measured samples in Fig. 3(c,d) are indicated by dots. Intuitively, a zero air-filling ratio results in a poor impedance match at the air/AAO interface, while an air-filling ratio near unity has a large reflection, similar to a pure aluminum surface. From Fig. 4, it can be observed that the sample 4 may have the optimum absorption property within the wavelength range of interest, which is consistent with the experimentally measured absorption spectrum in Fig. 3(c,d). We should note that the AAO films synthesized here have relatively large imaginary-part refractive indices such that a sufficiently thick AAO film may considerably absorb the impinging light in a relatively short optical path length. As a result, the surface-anodizing of lossy 6061-T6 aluminum may offer an effectual platform to tune the absorption and reflection properties of the AAO surface coating, of which the morphology, porosity and thickness can be readily controlled by varying the temperature, electrolyte concentration, and the DC bias in the anodizing process.

### Photothermal Effect

We investigate here the photothermal conversion efficiency of fabricated samples under excitation from a tungsten halogen illumination source, typically used to simulate the solar radiation in experiments. All samples to be characterized were insulated to avoid possible thermal conduction and convection, except for the surface to be illuminated. The intensity of impinging light is 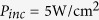. Figure 5(a) reports the transient temperature variation for different samples in Fig. 3(c), showing that the photothermal conversion efficiency is proportional to the absorption of the sample. The temperature was measured directly using K-type thermocouples connected to a temperature logger, with 0.1 K accuracy. It is astonishing to see that the steady-state temperature difference between an untreated aluminum and the sample 4 is more than 100 °C. Consider the balance of the energy supplied by the light-induced heat through phonon relaxations *Q*_I_ and the heat dissipation to external environment *Q*_ext_, one can obtain a relationship: 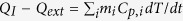, where *m*_i_ and *C*_p,I_ are mass and heat capacity of the *i*-th components of the system, *T* is temperature, and *t* is time. The heat energy supplied by the impinging light *Q*_I_ depends on several factors, including the optical absorption in materials, the intensity of light, and the fraction of light energy converted into thermal energy. In a linear thermal system, the rate of energy flowing out of the system is given by 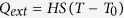, where *H* is the heat (dissipation) transfer coefficient, *S* is the exposure area, and *T*_0_ is the ambient temperature (here *T*_0_ = 30 °C, i.e. room temperature). For this initial condition problem, the time-dependent temperature variation of the absorber *T*(*t*) can be analyzed by the theoretical model^53^:


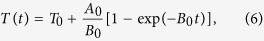


where 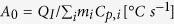 is the rate of energy adsorption, and


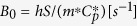
 is the rate constant of heat loss, *m* and *C*_p_ are effective mass and heat capacity of the background medium. We used Equation (6) to fit the temperature profiles in Fig. 5(a), and the extracted empirical parameters *A*_0_ and *B*_0_ are shown in Fig. 5(b), which are plotted as functions of the averaged spectral absorption. It is clearly seen that the measured temperature profile is perfectly described by the physics-based model of Equation (6), and the steady-state temperature can be estimated as 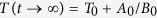, where taking the limit 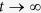 means the steady-state condition. Figure 5(b) shows the linear relationship between the energy absorption/dissipation rate and the optical absorption.

### Thermoelectronic Conversion

**Figure 1:**
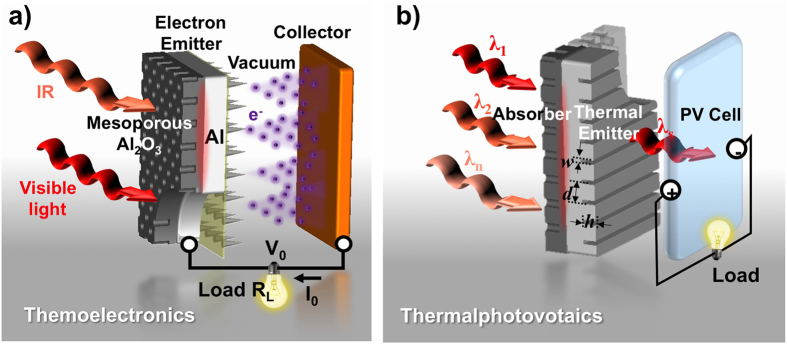
Schematics of (**a**) a thermoelectronic and (**b**) thermophotovoltaic microdevices using the mirror-backed nanoporous alumina absorber, which can be readily integrated with an electron or thermal emitter depending on applications.

We study here the applicability of this mirror-backed MMO structure to practical TC and TPV applications. We first consider a thermoelectronic device in Fig. 1(a), where the emitter electrode is composed of an AAO-coated aluminate sheet. The front-side AAO layer can absorb sunlight and/or blackbody radiation over a broad spectrum, converting absorbed photon energy into heat. The heated back-side metal, if coated with low-Schottky-barrier thermionic emitters will trigger the thermionic emission of hot electrons. Some low-dimensional nanomaterials are idea thermionic emitters, which show high thermal conductivity and low effective work function (e.g. LaB6 nanowires^54^, carbon nanotubes^55^, and graphene flakes^56^) or negative electron affinity (e.g. diamond nanotips^57^). When the vacuum gap between two electrodes is at the micrometer scale, one could neglect the space charge effect that limits the maximum current density received by the collector electrode^58^. The thermionic emission current density can be described by the well-known Richardson-Dushman formula as^9^:


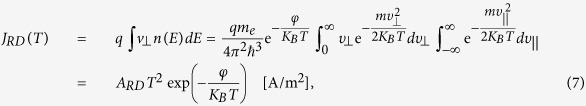


where 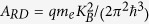 is the Richardson-Dushman constant, *n*(*E*) is the electron density of state, *E* is energy, *q* is the electron charge, *m*_e_ is the electron mass, *ħ* and *K*_B_ are the reduced Plank’s and Boltzmann’s constants, *φ* is the potential barrier at the metal surface [eV], and 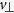 and 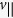 are electron velocity components normal and parallel to the metal surface. The net flux of electrons between the hot emitter with temperature *T*_e_ and the cold collector with temperature *T*_c_ is given by the difference between the forward current 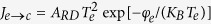 and the reverse current 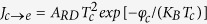, as 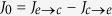. Previous works^8^,^9^,^59^ suggest that the work function of emitter electrode *Φ*_e_ should be larger than that of collector electrode *Φ*_c_, which renders


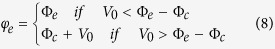



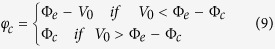


and *V*_0_ is the voltage drop at the load [see the inset of Fig. 6]. Also, heat is lost from transports of thermionically emitted electrons: 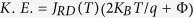. Although the ideal conversion efficiency neglecting heat losses, 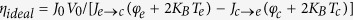, can approach 100% provided 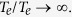 The practical efficiency is much lower due to unavoidable heat losses. According to the familiar Stefan–Boltzmann law^59^, the radiation heat loss is given by 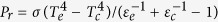, where *σ* is the Stefan–Boltzmann constant, *ε*_e_ and *ε*_c_ are emissivity of emitter and collector (here we assume 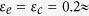 absorption of aluminum). Other possible heat transfer *P*_c_, e.g. thermal conduction loss in the proximity of collector and conversion loss of absorption to heat, is extracted from the measurement results in Fig. 5. The energy balance suggests the following relationship:


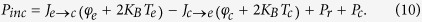


where *P*_*inc*_ is the illumination irradiance. Therefore, the optical-to-electrical conversion efficiency is defined as:


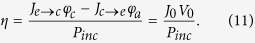


**Figure 2:**
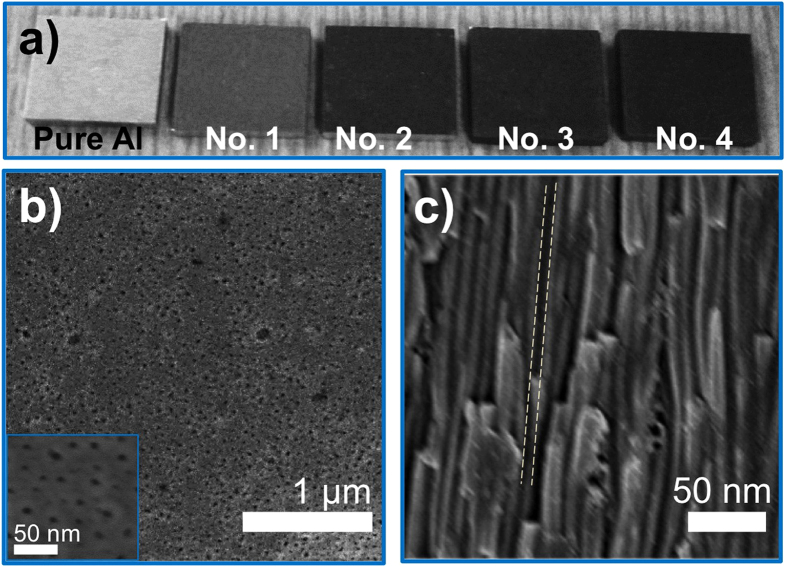
(**a**) CCD images for different species in Table 1. (**b**) Top-view and (**c**) cross-sectional SEM images for sample No. 4, showing irregularly-distributed, random-sized air nanopores.

Assuming the electrical contacts are perfect thermal insulator and perfectly electric conductor, the conversion efficiency can be calculated by simultaneously solving Equations (10) and (11). The optimum conversion efficiency is achieved at the voltage *V*_*m*_, which produces the maximum power, delivered to the load, namely 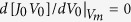
60. The value of *V*_*m*_ and the optimum conversion efficiency must be numerically obtained using an iterative method, analogous to the diagram of a photovoltaic cell^60^: performing an I–V sweep from short circuit to open circuit condition and recording the optimum operating point. Figure 6 presents the calculated theoretical maximum conversion efficiency against the intensity of impinging light for the different species shown in Fig. 2(a). We found that the surface anodizing can significantly boost the optical-to-electrical conversion efficiency of aluminum sheet at low illumination intensities, due to the enhanced light absorption and heat generation rate. We must emphasize that for the optimal design, i.e. sample 4, the device can be operated at a moderately low light intensity, readily achievable with a Fresnel lens. On the other hand, the untreated aluminum alloy requires an intense light ~25 W/cm^2^ to turn on the device. We note that by using specific thermodynamic structures, e.g. thermal metamaterials^61^, the thermal insulation and energy conversion efficiency (which in theory could be up to 30%^59^), could be improved.

### Thermophotovotaic Application

**Figure 3:**
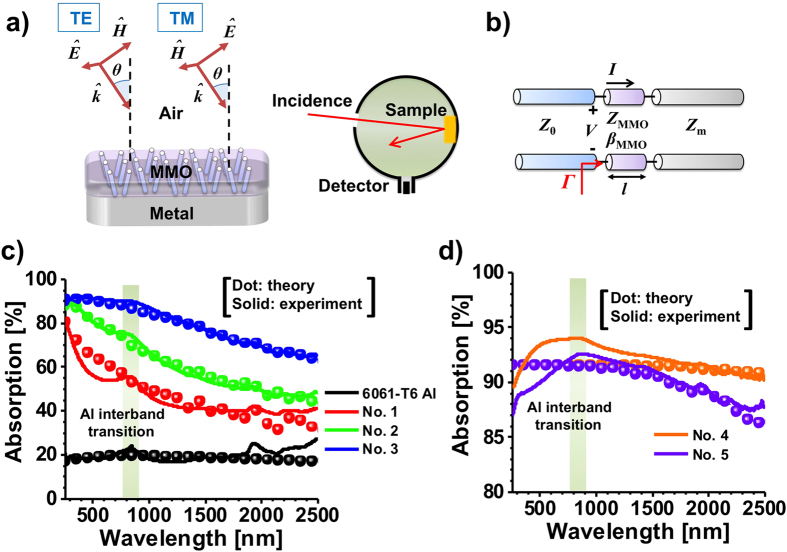
(**a**) Schematics of oblique incidence for TM- and TE-polarized plane waves, and reflection and absorption measurement based on UV-VIS-NIR spectrophotometer. (**b**) Corresponding transmission line model of (**a**). (**c**,**d**) are measured absorption against wavelength for pure 6061 T-6 aluminum sheet and different samples in Table 1; here solid lines represent the experimental data and dots represent the theoretical results based on the Maxwell-Garnet effective medium theory and transmission line approach.

**Figure 4:**
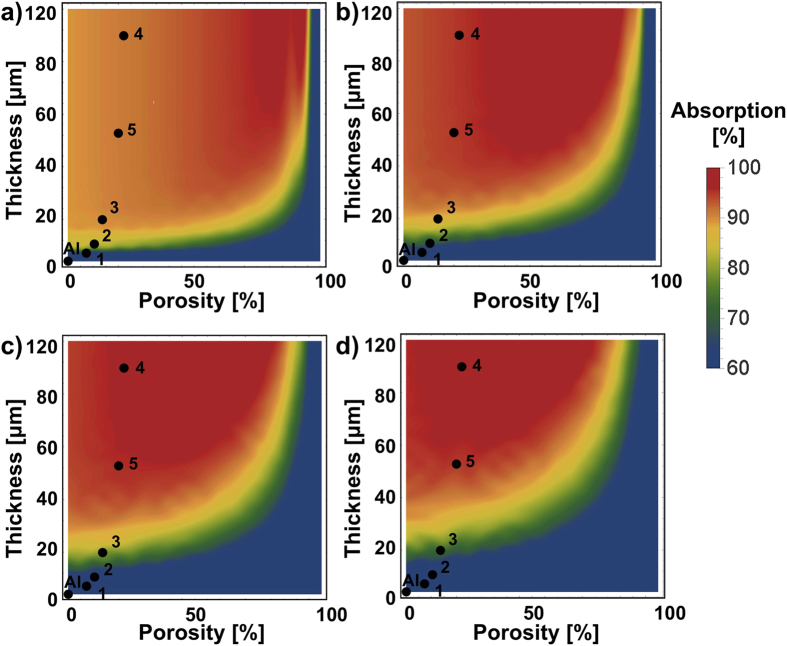
Theoretical contours of absorption for a mirror-backed porous alumina film, varying the porosity and thickness of alumina, at different wavelengths. (**a**) 500 nm, (**b**) 1000 nm, (**c**) 1500 nm and (**d**) 2000 nm. All samples in Fig. 3, with their corresponding characteristics summarized in Table 1, are indicated by dots.

**Figure 5:**
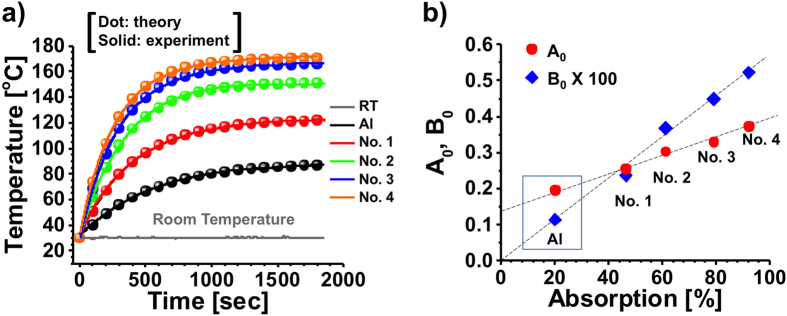
(**a**) Measured photothermal effect of temperature against time for different samples in Table 1 and the room temperature of reference. (**b**) Extracted energy adsorption rate and rate constant of heat loss from (**a**).

**Figure 6:**
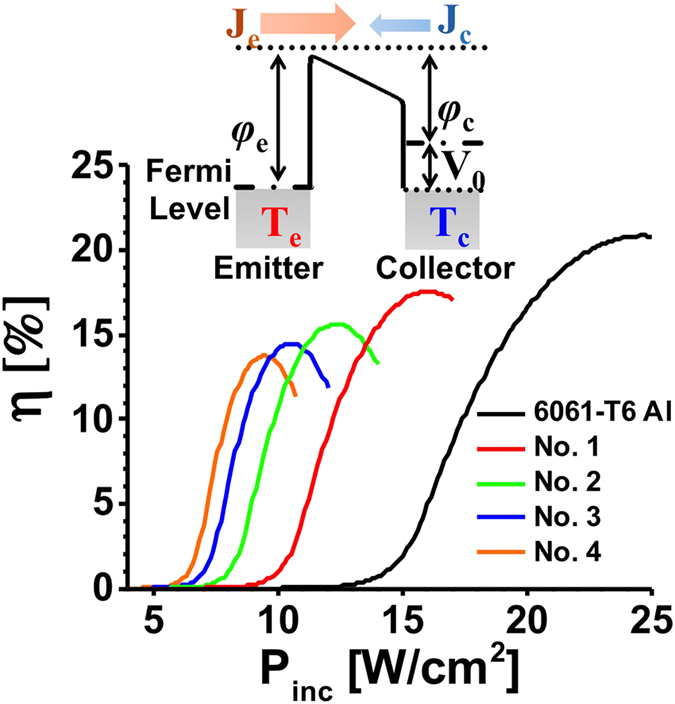
Predicted solar-to-electrical energy conversion efficiently against the illumination irradiance for different absorbers in Table 1; the inset shows the energy band diagram of a thermoelectronic microdiode with hot electronics thermionic emission.

We note that the proposed mirror-backed dark alumina structure can also be of interest for thermophotovotaic applications. The TPV device in Fig. 1(b) is known as a highly efficient tool to generate electricity. In this case, the front-side nanoporous alumina layer can collect light in a wide wavelength range, while the back-side metal can be nano-engineered to reradiate light in a narrow wavelength range, matched to the bandgap energy of photovoltaic diode receivers. In this scenario, nanostructures on each side of the metal substrate can be designed to tame the spectrum of absorption and reradiation. We note that the recently proposed metamaterial approach^62^,^63^,^64^ may be suitable here for the wide-angle, wavelength-selective thermal radiation. Figure 1(b) illustrates the proposed metamaterial structure, where a metamaterial slab is formed by a screen of thickness *h*, corrugated by slit of width *w* and period *d*. Provided the grating periodicity is subwavelength (*d* < *λ*/2), all diffraction orders, except for the zero-*th* mode, are evanescent, and therefore the TL approach in Fig. 3(b) can again be used to analyze the scattering properties. The homogenization theory for this metamaterial structure is more complicated than the mesoporous binary mixture in Equation (2). The metallic grating can be viewed as an array of subwavelength metal-air-metal waveguide that supports the fundamental TM mode (non-polarization-selective emitters are also possible by engineering the symmetric nanopatterns on the aluminum surface, as has been discussed in^26^). For a TM-wave illumination, the characteristic impedance per unit length is 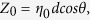 calculated as the ratio between the voltage across one period 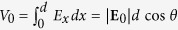 and the current per unit length 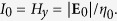 Inside each slit the modal propagation is independent of the incidence angle, with the complex wave vector *β*_s_, which satisfies the transcendental equation^62^:


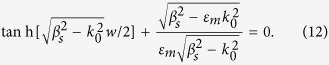


The characteristic impedance *Z*_s_ is defined as


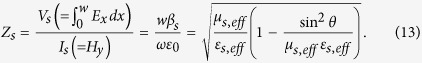


The momentum conservation for a homogeneous slab suggests


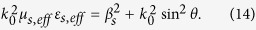


From Eqs. (13) and (14), the explicit expression for the effective material properties of metamaterial can be derived as:


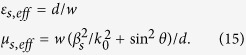


**Figure 7:**
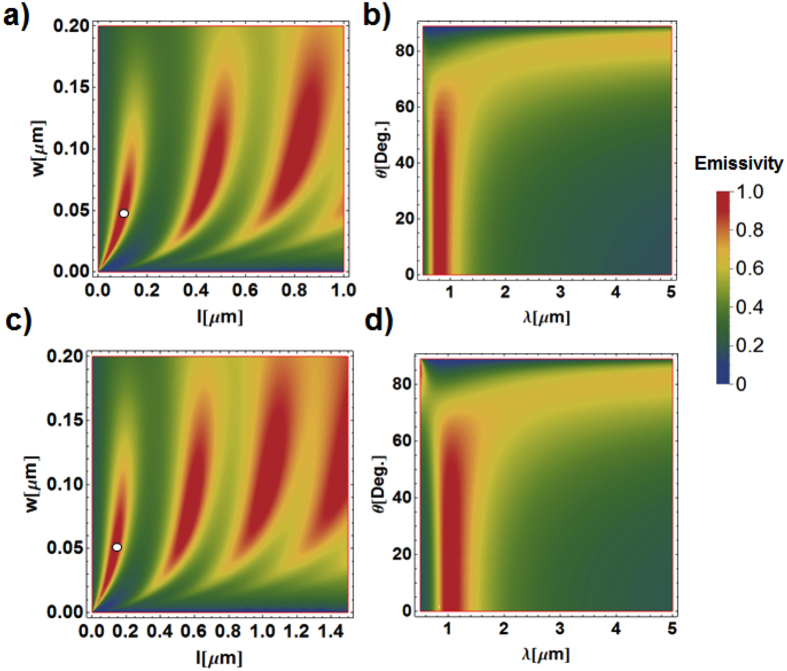
(**a**) Contours of emissivity for a metamaterial-based thermal emitter in Fig. 1(b), varying the width w and length h of nanoslits; here the period is d = 250 nm and the design wavelength is λ = 0.8 μm. (**b**) Contours of emissivity for a metamaterial-based thermal emitter using structural parameters indicated in the spot of (**a**), varying the wavelength and incident angle. (**c**,**d**) are similar to (**a**,**b**), but for the design wavelength λ = 1 μm.

Now, the scattering problem can be solved using the TL approach, analogous to Fig. 3(b), and the expressions for reflection and absorption are similar to Equations (3) and (4), by using the newly defined characteristic impedances and replacing *β*_MMO_ with 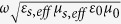 and *Z*_MMO_ with 
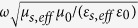
. Figure 7(a) reports the contour of emissivity (or absorption) calculated according to Equation (4) at the wavelength *λ* = 800 nm under the normal incidence (*θ* = 0°), varying the width and length (*w*, *h*) of air slits (here *d* = 250 nm). Bands of emissivity, with maximum value close to unity, are clearly visible in this figure. Figure 7(b) reports the contour of emissivity for a metamaterial using particularly structural parameter indicated in Fig. 7(a), varying wavelength and incident angle; here (*w*, *h*) = (50 nm, 100 nm). We found that the emissivity is fairly intense around the design wavelength *λ* = 800 nm, over a broad angular range. Figure 7(c,d) are similar to Fig. 7(b,c), but for a design wavelength of 1 μm, with structural parameters (*d*, *w*, *h*) = (250 nm, 50 nm, 100 nm), as indicated by the spot in Fig. 7(c). It is clearly seen that by tailoring the metamaterial geometry, narrowband emissivities can be designed at the desired wavelength range. This metamaterial approach may offer a worthwhile alternative to tame the thermal emissivity of back-side metal.

## Discussion

In summary, we have developed a mirror-backed *dark* alumina as an absorbing and photothermal conversion platform for harvesting sunlight and Earth’s infrared emissions. In particular, the nanoporous alumina made from impurity-rich aluminum via the electrochemical anodization can be optically lossy, while having a real-valued optical impedance matched to that of the free space. By optimizing the process conditions, the anodized aluminum turns completely dark, whereas the untreated aluminum shows brilliant reflection. The photothermal experiment further demonstrates the viability of proposed absorber in practical energy conversion applications. Finally, we have also theoretically investigated the conversion efficiency of a thermoelectronic solar cell based on the proposed absorber, showing a remarkably improved efficiency compared to an aluminum hot electrode. In addition, by integrating nanostructured metamaterials on the back-side of proposed absorber, the absorbed photon energy can produce a narrowband thermal emission to illuminate the PV module of thermophotovoltaic solar cells. The large-area and low-cost dark alumina shows a promising potential for various energy harvesting and conversion applications.

## Additional Information

**How to cite this article**: Farhat, M. *et al.* Mirror-backed Dark Alumina: A Nearly Perfect Absorber for Thermoelectronics and Thermophotovotaics. *Sci. Rep.* **6**, 19984; doi: 10.1038/srep19984 (2016).

## Figures and Tables

**Table 1:** Summary of physical properties for mirror-back nanoporous alumina absorbers prepared by hard anodizing with different process temperatures and applied voltages.

Sample No.	Process temperature [°C]	Process Voltage [V]	Air-filling ratio [%]	Thickness [μm]	A_0_[°Cs^−1^]	B_0_[s^−1^]
1	0	30	7	3.8	0.236	0.0025
2	5	30	10	7.8	0.368	0.0030
3	10	30	13	19	0.449	0.0033
4	10	50	21	103	0.522	0.0037
5	10	40	19	58.5	0.467	0.0032
6061-T6 Al	—	—	0	0	0.114	0.0019
